# Bile Acid Metabolism Affects Muscle Regeneration in Aging Skeletal Muscle in a Manner Associated with Regulation of ABCB1 Expression

**DOI:** 10.3390/ijms27062649

**Published:** 2026-03-13

**Authors:** Xiaoqing Wu, Yanan Wei, Qian Xue, Xia Li, Lihua Deng, Menghan Li, Yulan Liu, Jingtong Wang

**Affiliations:** 1 Gastroenterology Department, Peking University People’s Hospital, Beijing 100044, China; wuxq0611@163.com (X.W.);; 2 Geriatric Department, Peking University People’s Hospital, Beijing 100044, China; weiyanan@bjmu.edu.cn (Y.W.);

**Keywords:** sarcopenia, bile acid metabolism, muscle microvascular endothelial cells, ABCB1 transporter, skeletal muscle microenvironment

## Abstract

The role of bile acid metabolism within the skeletal muscle microenvironment in sarcopenia remains unclear. This study investigated bile acid alterations and the function of the ATP Binding Cassette Subfamily B Member 1 (ABCB1) transporter in muscle microvascular endothelial cells (MMECs) during aging. Using a sarcopenic mouse model stratified by muscle density, we found elevated deoxycholic acid (DCA) and lithocholic acid (LCA) levels but reduced tauroursodeoxycholic acid (TUDCA) levels in muscle, correlating with downregulated ABCB1/P-glycoprotein expression. In vitro, inhibition of ABCB1 in MMECs impaired bile acid efflux, promoted inflammation, and compromised endothelial health. Conditioned medium from these MMECs reduced the viability, proliferation, and differentiation of C2C12 myoblasts, downregulated myogenic factors, and increased atrophy markers. Furthermore, we identified miR-135a-5p as a direct upstream regulator of ABCB1 in MMECs, and demonstrated that it mediates bile acid efflux impairment and subsequent myoblast dysfunction. Our findings reveal a novel “bile acid–MMEC–muscle” axis in sarcopenia, where miR-135a-5p-mediated ABCB1 downregulation in MMECs disrupts the local bile acid milieu and impairs muscle regeneration, highlighting ABCB1 as a potential therapeutic target for aging-related muscle loss.

## 1. Introduction

Sarcopenia, a progressive, systemic skeletal muscle disorder, is characterized by loss of muscle mass, reduced muscle strength, and declined physical performance [1,2]. It is closely associated with adverse prognoses in various diseases, patient disability, and increased healthcare expenditures. Sarcopenic individuals typically face higher risks of adverse health events [3,4]. Globally, sarcopenia affects 10% to 16% of the elderly population [5]. As the population aged ≥60 years continues to grow, the number of individuals susceptible to sarcopenia is increasing [6]. With its prevalence rising annually, sarcopenia is projected to affect up to 23% of the elderly by 2100 [7]. However, to date, no Food and Drug Administration (FDA)-approved drugs exist to treat sarcopenia [8]. Therefore, further elucidating its pathogenesis, implementing effective preventive measures, and developing novel therapeutic approaches are critically important.

Current research on mechanisms underlying aging-associated sarcopenia includes impaired nutrient absorption [9], chronic inflammation [10], dysregulated autophagy [11], oxidative stress [12], mitochondrial dysfunction [13], and declined function of skeletal muscle satellite cells (SCs) [14]. Recently, the “gut–muscle axis” has emerged as a key research focus [15]. Notably, alterations in gut microbiota drive changes in bile acid metabolism, subsequently impacting skeletal muscle function [15,16,17]. Nevertheless, in mechanistic studies investigating this pathway, results regarding myofiber alterations induced by deoxycholic acid (DCA) and lithocholic acid (LCA) through TGR5 (G protein-coupled bile acid receptor 1)-mediated pathways remain inconsistent [18,19]; ursodeoxycholic acid (UDCA) interventions examining effects on muscle mass, strength, and quality also show inconsistent outcomes [20,21].

The ATP-binding cassette (ABC) transporter superfamily represents one of the largest and most functionally diverse groups of transmembrane proteins, utilizing ATP hydrolysis to facilitate the active transport of various substrates across biological membranes [22]. Among these, the ATP Binding Cassette Subfamily B Member 1 (ABCB1) gene (also known as Multidrug Resistance Protein 1 (MDR1)) encodes the P-glycoprotein (P-gp), a well-characterized multidrug efflux pump [23]. Historically recognized for its role in conferring multidrug resistance in cancer cells, P-gp is also constitutively expressed in normal tissues with barrier functions, such as the intestinal epithelium, hepatocytes, and blood–tissue barriers, where it plays a pivotal role in the excretion of xenobiotics and endogenous metabolites, including specific bile acids [20,24].

Notably, within the skeletal muscle microenvironment, ABCB1 is predominantly localized on the membrane of microvascular endothelial cells, where it functions as a critical gatekeeper for metabolite exchange between the systemic circulation and the muscle interstitium. The vascular endothelium constitutes a critical component of the vascular system, responsible for maintaining vascular tone, angiogenesis, and hemostatic functions while concurrently exhibiting antioxidant, anti-inflammatory, and antithrombotic properties [25]. Capillary endothelial dysfunction may precipitate vascular complications and consequently plays a pivotal role in various cardiovascular disorders [26] and metabolism-related diseases, including type 2 diabetes mellitus (T2DM) [27], obesity [28], and hypercholesterolemia [29,30]. Skeletal muscle microvascular endothelial cells (MMECs) serve as essential intermediaries facilitating communication between the skeletal muscle microenvironment and external milieu. As a critical part of the skeletal muscle microvascular unit (MVU), the endothelial cells are primarily involved in the regulation of the perfusion and convective transport of substrates [31]. Additionally, they are also responsible of the leukocyte recruitment in the inflammatory processes [32]. Studies have indicated that dysfunction of MMECs is associated with the development of myopathies [32,33]. Evidence suggests concurrent manifestation of skeletal muscle atrophy and endothelial dysfunction in numerous chronic diseases.

While the systemic circulation of gut-derived bile acids is well-documented, their specific influx and regulatory effects within the peripheral skeletal muscle microenvironment remain poorly defined. We hypothesize that dysregulation of the local bile acid pool is not merely a consequence of systemic metabolic shifts, but is actively modulated by transport mechanisms within MMECs, which in turn profoundly dictates the skeletal muscle microenvironment and regenerative capacity.

Therefore, this study aims to comprehensively explore alterations in bile acid concentrations within the skeletal muscle microenvironment and their association with ABCB1 transporter functionality in MMECs, while evaluating the consequent effects of these changes on muscle mass and functional capacity.

## 2. Results

### 2.1. Altered Bile Acid Concentrations and Abcb1 Expression in Sarcopenic Muscle

To investigate the role of bile acid metabolism in sarcopenia, we employed naturally aged *C57BL/6J* mice (24-month-old, n = 16) as the sarcopenic model and young mice (4-month-old, n = 8) as the Control Group. To account for the heterogeneity of aging, the sarcopenic mice were further stratified into Sarcopenia Group A (n = 8) and Sarcopenia Group B (n = 8) based on the median value (119.62 HU) of Skeletal Muscle Density (SMD), to reduce the confounding effects of age. SMD was defined as the mean Hounsfield Unit (HU) value of the skeletal muscle, representing a key metric for muscle quality. Grip duration, mean grip strength, and grip strength-to-body weight ratio differed significantly among Sarcopenia Group A, Sarcopenia Group B, and the Control Group (*p* < 0.002). Compared to the Control Group, both Sarcopenia Group A and Sarcopenia Group B exhibited reduced grip duration and significantly decreased mean grip strength and grip strength-to-body weight ratio, with more pronounced reductions in Sarcopenia Group B (Table 1). These functional declines demonstrate the validity of the sarcopenic mouse model and the appropriateness of group stratification. Moreover, gastrocnemius muscle was selected for analysis as it is easily obtained, critical in lower limb activities, susceptible to aging-related atrophy in wild-type mice, and is regarded as a good representative of skeletal muscle mass and strength in sarcopenia mouse models [34]. Conversely, no significant intergroup differences were observed in gastrocnemius muscle weight (*p* = 0.071) or gastrocnemius-to-body weight ratio (*p* = 0.197).

**Figure 1:**
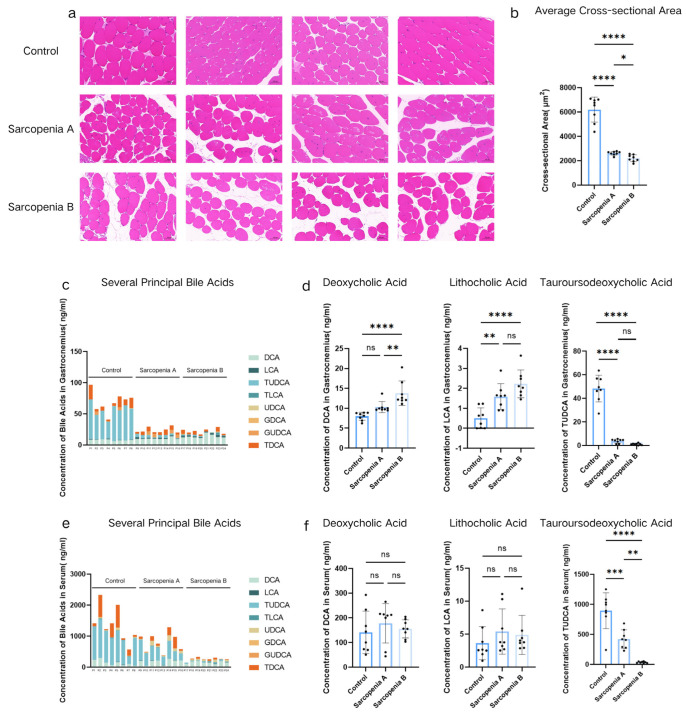
Differences in cross-sectional area (CSA) and Concentrations of Secondary Bile Acids in Serum and Muscle Tissue Microenvironment Among the Control Group, Sarcopenia Group A, and Sarcopenia Group B. Representative H&E-stained cross-sections of gastrocnemius muscle from the Control Group, Sarcopenia Group A, and Sarcopenia Group B mice (**a**) (scale bar: 50 μm), and CSA differences (**b**). Stacked bar charts displaying the composition and concentration of several principal bile acids in the gastrocnemius muscle microenvironment (**c**) and serum (**e**). In these stacked bar charts (**c**,**e**), each individual bar represents the comprehensive bile acid profile of a single mouse. Individual bar charts comparing the specific concentrations of deoxycholic acid (DCA), lithocholic acid (LCA), and tauroursodeoxycholic acid (TUDCA) in the gastrocnemius muscle microenvironment (**d**) and serum (**f**). In these individual bar charts (**d**,**f**), the height of each bar represents the mean concentration of the respective group, with error bars indicating the standard deviation (SD). The dots overlaid on the bars represent distinct biological replicates (n = 8 per group). All bile acid concentrations were measured by high-performance liquid chromatography–tandem mass spectrometry (HPLC-MS/MS). * *p* < 0.05; ** *p* < 0.01; *** *p* < 0.001; and **** *p* < 0.0001; *p* > 0.05 was considered as not significant (ns).

As shown in Figure 1a,b, histological evaluation of H&E-stained skeletal muscle sections revealed reduced fiber density and increased fat infiltration in the sarcopenic groups versus the Control Group. Concurrently, closer examination of the individual remaining myofibers revealed profound morphological atrophy (highly magnified representative fields demonstrating this individual cellular atrophy are provided in Supplementary Figure S1). To precisely quantify these microstructural alterations, we utilized SlideViewer software (version 2.5) to measure the total cross-sectional area (CSA) and record the fiber count across multiple randomized microscopic fields; the mean CSA was subsequently calculated by dividing the total area by the number of fibers. Statistical analyses of these calculated values confirmed a significantly larger mean CSA in the Control Group than in Sarcopenia Group A (*p* < 0.0001) and Sarcopenia Group B (*p* < 0.0001), with a significant intergroup disparity between the two sarcopenic groups (*p* = 0.0132). To precisely investigate bile acid impacts on skeletal muscle, we quantified major bile acids in both the skeletal muscle microenvironment and serum. The comprehensive composition profiles of several principal bile acids are presented in Figure 1c (muscle) and Figure 1e (serum). Based on prior evidence [19,35], we focused on secondary bile acids critically involved in systemic metabolism—DCA, LCA, and TUDCA—the latter being known to influence muscle tissue [20]. Figure 1d demonstrates significantly elevated microenvironmental DCA in Sarcopenia Group B versus both the Control Group (*p* < 0.0001) and Sarcopenia Group A (*p* = 0.0069). Similarly, LCA increased in Sarcopenia Group A (*p* = 0.0060) and Sarcopenia Group B (*p* < 0.0001) relative to the Control Group. Conversely, TUDCA decreased substantially in both sarcopenic groups versus controls (*p* < 0.0001 each), though without significant differences between the two sarcopenic groups (Figure 1d). However, only the concentration of TUDCA in serum differed among the three groups; no significant differences were observed in DCA and LCA (Figure 1f).

Analysis of the remaining bile acids in mouse gastrocnemius muscle revealed that taurocholic acid (TCA) concentration showed a significant difference between the Control Group and Sarcopenia Group B (*p* = 0.0343). TCA concentration was elevated in the gastrocnemius muscle of Sarcopenia Group B. Tauro-α-muricholic acid (T-α-MCA) (*p* = 0.0435) and tauro-β-muricholic acid (T-β-MCA) (*p* = 0.0418) both exhibited significantly reduced concentrations in the gastrocnemius muscle of Sarcopenia Group B. Furthermore, compared with the Control Group, glycocholic acid (GCA) concentration was also decreased in the gastrocnemius muscle of Sarcopenia Group B, with a statistically significant difference (*p* = 0.0128) (Supplementary Figure S2).

**Figure 2:**
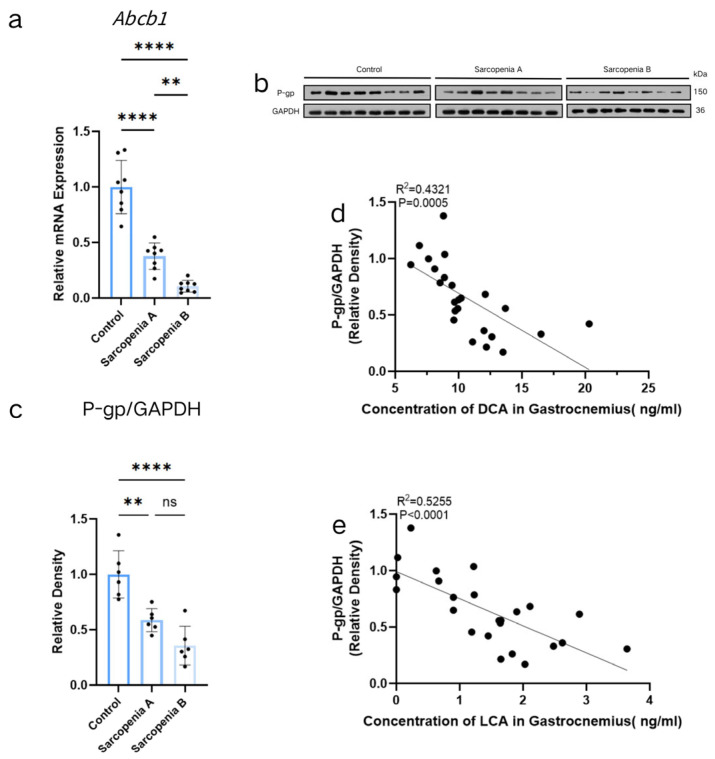
Changes in *ATP Binding Cassette Subfamily B Member 1 (Abcb1)* Expression in Gastrocnemius Muscle and Correlations with DCA and LCA Concentrations in the Tissue Microenvironment of the Control Group, Sarcopenia Group A, and Sarcopenia Group B. *Abcb1* mRNA levels in mouse gastrocnemius muscle were measured by quantitative reverse transcription polymerase chain reaction (qRT-PCR) (**a**). Representative Western blot images of P-gp and GAPDH proteins. For each target, lanes 1–8: Control Group; lanes 9–16: Sarcopenia Group A; and lanes 17–24: Sarcopenia Group B (**b**). Quantitative analysis of P-gp protein expression levels normalized to GAPDH (n = 8 per group) (**c**). Spearman’s bivariate correlation analysis was performed to assess correlations between DCA/LCA concentrations in the skeletal muscle microenvironment and P-gp expression (**d**,**e**). ** *p* < 0.01; **** *p* < 0.0001, *p* > 0.05 was considered as not significant (ns).

Given that P-gp, encoded by the *Abcb1* gene, functions as a critical ATP-dependent efflux pump capable of transporting endogenous metabolites including bile acids, we hypothesized that its dysregulation might drive the observed bile acid accumulation. To rigorously test whether this transport impairment was specific to P-gp, we additionally evaluated the mRNA expression of other major systemic and peripheral bile acid/multidrug efflux transporters, including the bile salt export pump (BSEP) and multidrug resistance-associated proteins 1-4 (MRP1-4). Notably, qRT-PCR analysis revealed no significant differences in the expression of *Bsep* or *Mrp1-4* among the Control, Sarcopenia A, and Sarcopenia B groups (Supplementary Figure S3). In stark contrast, we found that *Abcb1* mRNA expression was significantly reduced in the muscle tissues of both Sarcopenia Group A (*p* < 0.0001) and Sarcopenia Group B (*p* < 0.0001) compared with the Control Group. Furthermore, compared with Sarcopenia Group A, *Abcb1* mRNA expression was significantly decreased in Sarcopenia Group B (*p* = 0.0067) (Figure 2a). Western blot analysis demonstrated that P-gp protein expression was significantly downregulated in the muscles of the both Sarcopenia Group A (*p* = 0.0021) and Sarcopenia Group B compared with the Control Group (*p* < 0.0001), while no significant difference was observed between Sarcopenia Group B and Sarcopenia Group A (*p* > 0.05) (Figure 2b,c). As shown in Figure 2d,e, Spearman’s bivariate correlation analysis indicated that DCA concentration in the muscle tissue microenvironment exhibited a significant negative correlation with *p*-gp expression (*p* = 0.0005). Additionally, LCA concentration showed a significant negative correlation with P-gp expression (*p* < 0.0001), with correlation coefficients of −0.6574 and −0.7249, respectively.

To elucidate the specific role of *Abcb1* in mediating bile acid transport and muscle regeneration, we performed in situ muscle injections in sarcopenic mice with adeno-associated virus serotype 9 (AAV9)-oe-*Abcb1* vectors or their corresponding negative controls. The vectors were delivered via multipoint injection into the right gastrocnemius muscle, and the therapeutic effects were evaluated four weeks post injection. The overexpression efficiency was validated by Western blot and qRT-PCR analysis (*p* < 0.001) (Supplementary Figure S4a,b). The results of H&E staining of skeletal muscle fibers showed increased CSA and elevated fiber density by overexpressing *Abcb1* compared with the Sarcopenia + oe-NC group, suggesting the effects of *Abcb1* in promoting muscle regeneration in sarcopenic mice (Supplementary Figure S4c). Moreover, we investigated the effects of overexpressing *Abcb1* on bile acid metabolism in skeletal muscle tissue and serum. The results indicated that the concentration of DCA and LCA in the Sarcopenia + oe-ABCB1 group was elevated compared to the Sarcopenia + oe-NC group (*p* < 0.0001), while their concentration in mouse serum was not significantly altered across groups (*p* > 0.05). Notably, this substantial increase in TUDCA was observed consistently across both the local gastrocnemius muscle microenvironment and the systemic circulation (Supplementary Figure S4d,e). Collectively, these findings demonstrate that AAV9-mediated *Abcb1* overexpression significantly modulates the bile acid profile, promoting a systemic enrichment of the cytoprotective TUDCA.

### 2.2. Correlation of Specific Bile Acids in the Microenvironment with Skeletal Muscle Mass and Function

**Figure 3:**
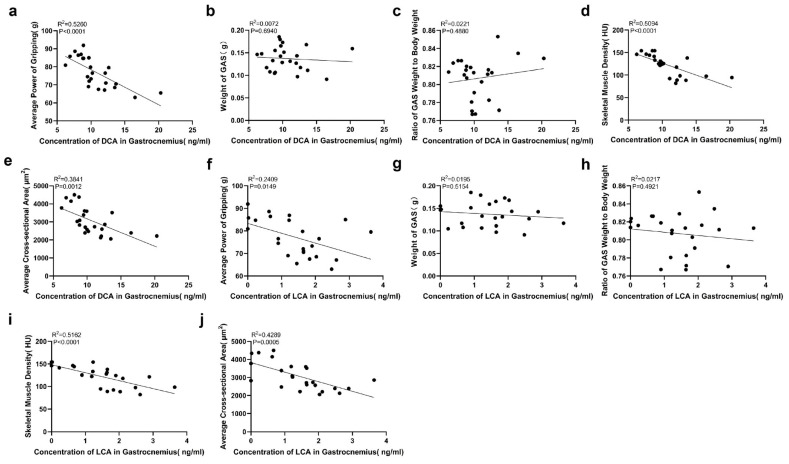
Correlation Analyses Between DCA and LCA Concentrations in Mouse Skeletal Muscle and Grip Strength, Unilateral Gastrocnemius Weight, Unilateral Gastrocnemius Weight/Body Weight, Skeletal Muscle Density (SMD), and CSA. Spearman’s bivariate correlation analyses were performed to assess the correlations of DCA concentration in skeletal muscle with mean grip strength (**a**), unilateral gastrocnemius weight (**b**), unilateral gastrocnemius weight/body weight (**c**), SMD (**d**), and CSA (**e**). Similarly, correlations of LCA concentration in skeletal muscle with mean grip strength (**f**), unilateral gastrocnemius weight (**g**), unilateral gastrocnemius weight/body weight (**h**), SMD (**i**), and CSA (**j**) were analyzed.

Spearman’s bivariate correlation analysis was performed to investigate the correlations between DCA and LCA concentrations in the skeletal muscle tissue microenvironment and grip strength, unilateral gastrocnemius weight, unilateral gastrocnemius weight/body weight, SMD measured by micro-CT, and CSA in mice (Figure 3). The results demonstrate significant negative correlations between DCA concentration in skeletal muscle and mean grip strength (*p* < 0.0001), SMD (*p* < 0.0001), and CSA (*p* = 0.0012), with correlation coefficients of −0.7253, −0.7137, and −0.6198, respectively. Simultaneously, significant negative correlations were observed between LCA concentration in skeletal muscle and mean grip strength (*p* = 0.0149), SMD (*p* < 0.0001), and CSA (*p* = 0.0005), with correlation coefficients of −0.4908, −0.7185, and −0.6549, respectively.

### 2.3. Effects of Inhibiting or Enhancing Bile Acid Efflux on MMECs

**Figure 4:**
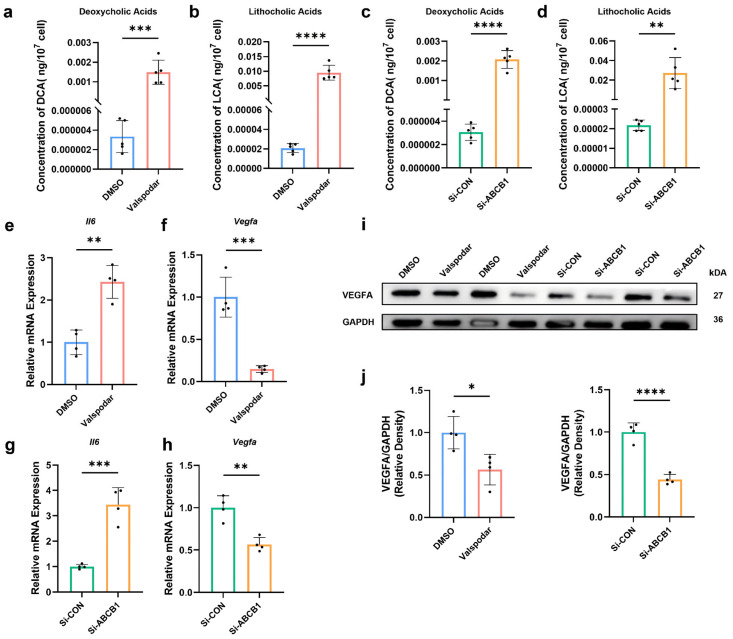
Increased Intracellular Bile Acid Concentrations and Altered Expression of Endothelial Function Regulatory Genes in Skeletal muscle microvascular endothelial cells (MMECs) of the Valspodar Group and Si-ABCB1 Group. Intracellular DCA (**a**,**c**) and LCA (**b**,**d**) concentrations in MMECs were measured by HPLC-MS/MS. Expression of *interleukin 6 (Il6)* (**e**,**g**) and *vascular endothelial growth factor A (Vegfa)* (**f**,**h**) in MMECs was detected by qRT-PCR. VEGFA protein expression levels were analyzed by Western blot with quantification (**i**,**j**). * *p* < 0.05; ** *p* < 0.01; *** *p* < 0.001; and **** *p* < 0.0001.

To investigate the functional role of ABCB1 in MMECs, we employed both pharmacological and genetic approaches. MMECs were treated with Valspodar (10 μM), a potent and selective P-glycoprotein (P-gp) inhibitor, with 0.008% DMSO serving as the vehicle control. For genetic knockdown, ABCB1-targeting small interfering RNA (si-ABCB1) was used, with scrambled siRNA (si-CON) as the negative control. After a 24 h pharmacological or 72 h genetic intervention, a bile acid mixture (DCA and LCA) was added to evaluate transport capacity. Compared with the DMSO Group and the Si-CON Group, intracellular DCA concentrations in MMECs were significantly reduced in the Valspodar Group (*p* = 0.0007) and the Si-ABCB1 Group (*p* < 0.0001). Similarly, intracellular LCA concentrations decreased significantly in the Valspodar Group (*p* < 0.0001) and the Si-ABCB1 Group (*p* = 0.0051) (Figure 4a–d). Compared with the DMSO Group and the Si-CON Group, gene expression of key endothelial regulatory proteins in MMECs was altered in the Valspodar Group and the Si-ABCB1 Group. Specifically, increased expression of *interleukin 6 (Il6)* (Figure 4e,g), alongside decreased expression of *vascular endothelial growth factor A (Vegfa)* (Figure 4f,h–j), was observed. Similarly, in our supplementary analyses, we also detected decreased expression of *endothelial nitric oxide synthase (Enos)*, and *superoxide dismutase 2 (Sod2)* (Supplementary Figure S5a,b,d,e), while the inflammatory factor *tumor necrosis factor-α (Tnf-α)* was upregulated (Supplementary Figure S5c,f).

### 2.4. Effects of Conditioned Medium (CM) from MMECs on C2C12 Cell Viability and Proliferation

**Figure 5:**
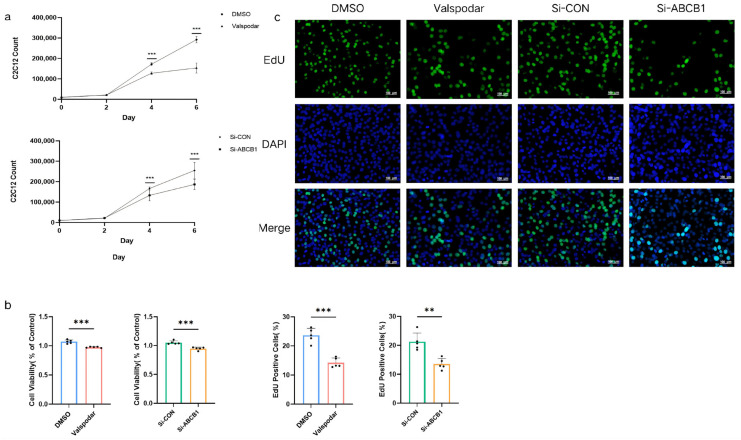
Effects of conditioned medium (CM) from Different MMEC Groups on C2C12 Viability and Proliferation. C2C12 counts in the Valspodar Group, the DMSO Group, the Si-ABCB1 Group, and the Si-CON Group (**a**). C2C12 cell viability assessed by CCK-8 assay across groups (**b**). Representative EdU and DAPI staining images with statistical analysis of proliferating (EdU^+^) C2C12 cell percentages (**c**). ** *p* < 0.01; *** *p* < 0.001.

C2C12 cells were treated with a 1:1 mixture of myoblast basal medium and CM from the Valspodar Group, the DMSO Group, the Si-ABCB1 Group, and the Si-CON Group of MMECs. From day 4 onward, fewer C2C12 cells were observed in the Valspodar Group and the Si-ABCB1 Group versus their respective controls (Figure 5a). A CCK-8 assay on day 4 revealed significantly reduced C2C12 viability in the Valspodar Group (*p* = 0.0002) and the Si-ABCB1 Group (*p* = 0.0004) compared to controls (Figure 5b). To further evaluate the impact of MMEC-derived conditioned medium on C2C12 proliferation, we performed an EdU incorporation assay. EdU, a thymidine analog that is incorporated into newly synthesized DNA, labels cells actively progressing through the S-phase of the cell cycle. On day 4, EdU and DAPI staining (Figure 5c) showed significantly lower proportions of EdU^+^ C2C12 cells in the Valspodar Group (*p* = 0.0001) and the Si-ABCB1 Group (*p* = 0.0015) versus the DMSO Group and the Si-CON Group.

### 2.5. Effects of CM from MMECs on Inflammatory Factor Expression and Differentiation-Related Functions in C2C12-Derived Myotubes

**Figure 6:**
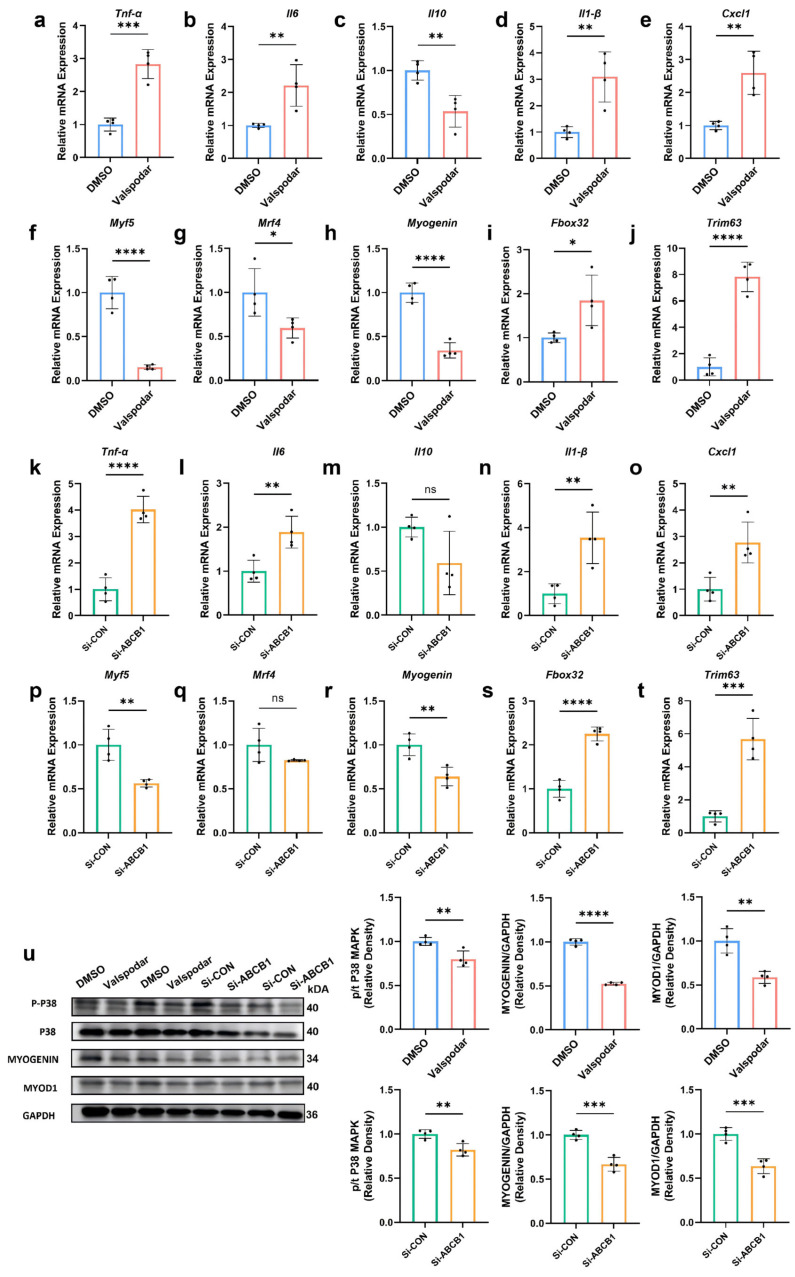
Effects of CM from Different MMEC Groups on Inflammatory Factors and Differentiation-Related Functions in C2C12-Derived Myotubes. Expression of inflammatory factors *Tumor necrosis factor-alpha (Tnf-α)* (**a**,**k**), *Interleukin 6 (Il6)* (**b**,**l**), *Interleukin 10 (Il10)* (**c**,**m**), *Interleukin 1β (Il1β)* (**d**,**n**), and *C-X-C motif chemokine ligand 1 (Cxcl1)* (**e**,**o**) in myotubes treated with CM from the Valspodar Group, the DMSO Group, the Si-ABCB1 Group, and the Si-CON Group of MMECs was detected by RT-qPCR. Expression of myogenic key genes *Myogenic factor 5* (Myf5) (**f**,**p**), *Muscle regulatory factor 4 (Mrf4)* (**g**,**q**), Myogenin (**h**,**r**), *F-box protein 32* (Fbxo32) (**i**,**s**), and *Tripartite motif containing 63 (Trim63)* (**j**,**t**) was also analyzed. Protein expression levels of phospho-p38 (p-p38), total p38 mitogen-activated protein kinase (p38 MAPK), MYOGENIN, and myogenic differentiation 1 (MYOD1) were assessed by Western blot with quantification (**u**). * *p* < 0.05; ** *p* < 0.01; *** *p* < 0.001; and **** *p* < 0.0001, *p* > 0.05 was considered as not significant (ns).

C2C12 myoblasts were treated with a 1:1 mixture of myogenic differentiation medium and CM from different MMEC groups, with daily medium changes for 6 days to obtain differentiated myotubes. We assessed inflammatory factor expression, differentiation-related proteins, and signaling pathways. Compared to respective controls, myotubes treated with CM from the Valspodar Group and the Si-ABCB1 Group exhibited significantly increased *Tnf-α*, *Il6*, *interleukin 1β (Il1β)*, and *C-X-C motif chemokine ligand 1 (Cxcl1)* expression alongside decreased *interleukin 10 (Il10)* expression (Figure 6a–e,k–o).

Expression of key myogenic genes *Myogenic factor 5 (Myf5)*, *Muscle regulatory factor 4 (Mrf4)*, and *Myogenin* was reduced in response to *Abcb1* silencing or inhibition via Valspodar (Figure 6f–h,p–r). Specifically, compared with the Si-CON Group, myotubes treated with CM from the Si-ABCB1 Group showed a decreasing trend in *Mrf4* expression (Figure 6q), though this difference was not statistically significant. Moreover, expression of atrophy-related genes *F-box protein 32 (Fbxo32)* and *Tripartite motif containing 63 (Trim63)* were significantly elevated in myotubes treated with CM from the Valspodar Group and the Si-ABCB1 Group (Figure 6i,j,s,t).

The p38 mitogen-activated protein kinase (p38 MAPK) pathway regulates myoblast growth and differentiation. Compared with respective controls, phospho-p38 MAPK levels were significantly reduced in myotubes treated with CM from the Valspodar Group (*p* = 0.0075) and the Si-ABCB1 Group (*p* = 0.0058) (Figure 6u). Subsequently, we validated protein-level differences in MYOGENIN and myogenic differentiation 1 (MYOD1). Both proteins showed significantly decreased expression in myotubes treated with CM from the Valspodar Group (*p* < 0.0001 for MYOGENIN; *p* = 0.0017 for MYOD1) and the Si-ABCB1 Group (*p* = 0.0004 for MYOGENIN; *p* = 0.0006 for MYOD1) versus the DMSO Group and the Si-CON Group (Figure 6u).

### 2.6. Effects of CM from Valspodar- and Si-ABCB1-Treated MMECs on Myotube Diameter, Area, and Fusion

**Figure 7:**
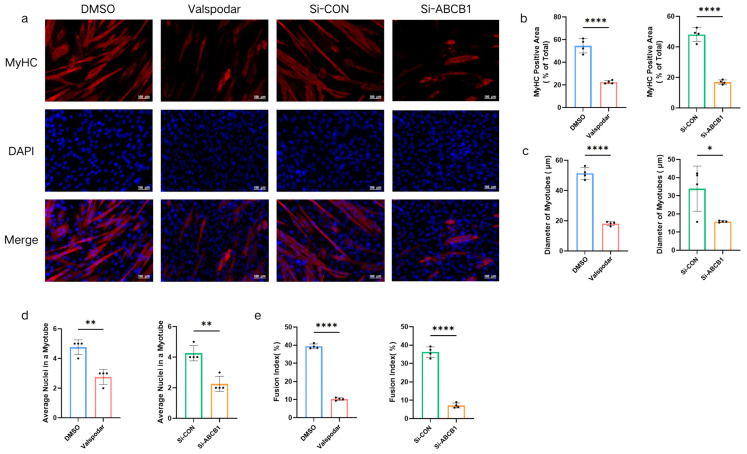
Immunofluorescence Staining of C2C12-Derived Myotubes Treated with CM from Different MMEC Groups. Representative images of Myosin Heavy Chain (MyHC) and DAPI staining (**a**). Scale bar = 100 μm; red: MyHC, blue: nuclei. Measured and statistically analyzed: MyHC-positive area proportion (**b**), myotube diameter (**c**), average nuclei number per myotube (**d**), and myotube fusion index (**e**). * *p* < 0.05; ** *p* < 0.01; and **** *p* < 0.0001.

Myotube differentiation was assessed via DAPI and Myosin Heavy Chain (MyHC) staining (a late-stage myogenic marker), with representative images shown in Figure 7a. Compared with the DMSO Group and the Si-CON Group, myotubes treated with CM from the Valspodar Group and the Si-ABCB1 Group exhibited significantly lower MyHC-positive area proportions (*p* < 0.0001 for Valspodar; *p* < 0.0001 for Si-ABCB1) (Figure 7b), smaller diameters (*p* < 0.0001 for Valspodar; *p* = 0.0272 for Si-ABCB1) (Figure 7c), and shorter lengths. Myotubes treated with CM from the Valspodar Group and the Si-ABCB1 Group exhibited significantly fewer nuclei per myotube (*p* = 0.0013 for both groups) (Figure 7d) and significantly lower myotube fusion index scores (*p* < 0.0001 for both groups) (Figure 7e).

### 2.7. ABCB1 Is Targeted by miR-135a-5p in MMECs

**Figure 8:**
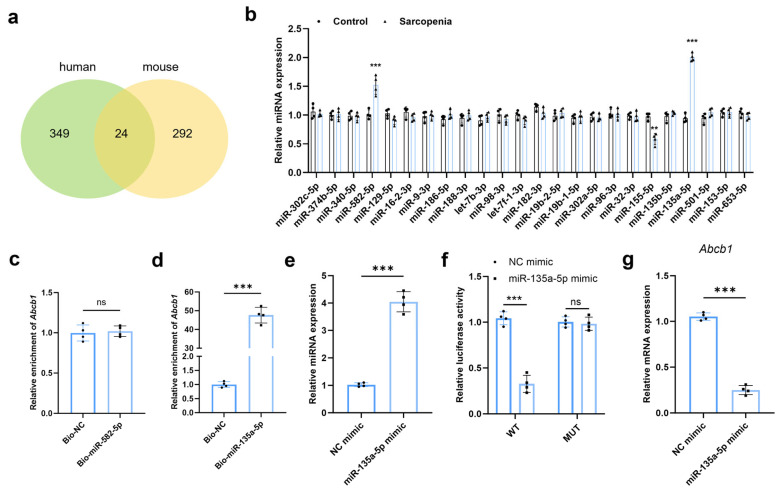
*Abcb1* was targeted by miR-135a-5p in MMECs. Candidate human and mouse miRNAs predicted to target ABCB1/*Abcb1* on Tagetscan database (**a**). Expression of screened miRNAs in muscle tissues from the Control Group and Sarcopenia Group (**b**). RNA pull-down assays were conducted to detect the binding between *Abcb1* and miR-582-5p (**c**) or miR-135a-5p (**d**) in MMECs. qRT-PCR was used to detect the overexpression efficiency of miR-135a-5p in MMECs (**e**). Luciferase reporter assays were performed to determine the interaction between miR-135a-5p and *Abcb1* in MMECs (**f**). qRT-PCR was used to detect the expression of *Abcb1* mRNA in MMECs transfected with NC mimics or miR-135a-5p mimics (**g**). ** *p* < 0.01; *** *p* < 0.001, *p* > 0.05 was considered as not significant (ns).

The potential upstream regulatory mechanism of ABCB1 was explored. MicroRNAs (miRNAs) are involved in various biological processes [35,36]. In this study, we investigated the potential miRNAs that regulated ABCB1 in MMECs. The candidate miRNAs for human and mouse *Abcb1* genes were searched on the Targetscan database (https://www.targetscan.org/vert_80/ (accessed on 10 January 2026)), and a total of 24 miRNAs were obtained after intersection (Figure 8a). To identify the universal epigenetic initiators driving the overall pathogenesis of sarcopenia, rather than stage-specific progression markers, the candidate miRNAs were screened across a unified Sarcopenia cohort (combining groups A and B) and compared to the healthy Control Group. The expression of those candidate miRNAs was measured in the muscle tissues from control and sarcopenic mice. The results showed that only two miRNAs (miR-582-5p and miR-135a-5p) were upregulated and one miRNA (miR-155-5p) was downregulated. As *Abcb1* was downregulated in sarcopenia, two upregulated miRNAs were likely to regulate its expression via direct binding and were selected for further analysis (Figure 8b). RNA pull-down assays were conducted, and the results indicate that ABCB1 was enriched in the complexes pulled down by bio-miR-135a-5p (*p* < 0.001) but not by bio-miR-582-5p (Figure 8c,d). We then overexpressed miR-135a-5p in MMECs and examined the overexpression efficacy using qRT-PCR analysis (*p* < 0.001) (Figure 8e). Luciferase reporter assays were conducted to verify the binding between miR-135a-5p and ABCB1. Consistently, the results showed that miR-135a-5p overexpression significantly decreased the luciferase reporter activity in the WT group (*p* < 0.001) but not the MUT group, validating the interaction between ABCB1 and miR-135a-5p (Figure 8f). Additionally, we measured the expression of ABCB1 in MMECs with miR-135a-5p overexpression, and qRT-PCR analysis exhibited that ABCB1 was significantly downregulated in MMECs transfected with miR-135a-5p mimics (*p* < 0.001) (Figure 8g).

### 2.8. MiR-135a-5p Inhibits Bile Acid Efflux of MMECs by Regulating ABCB1

**Figure 9:**
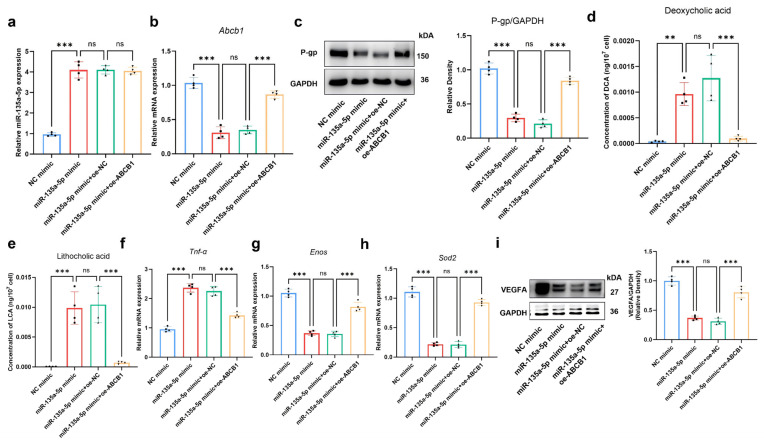
Effects of miR-135a-5p/*Abcb1* Axis on Bile Acid Efflux of MMECs. qRT-PCR analysis of miR-135a-5p (**a**,**b**) *Abcb1* mRNA expression in MMECs in each group. Western blot analysis and quantification of P-gp protein expression in MMECs (**c**). Intracellular DCA (**d**) and LCA (**e**) concentrations in MMECs were measured by HPLC-MS/MS. Expression of *Tnf-α* (**f**), *endothelial nitric oxide synthase (Enos)* (**g**), and *superoxide dismutase 2 (Sod2)* (**h**) in MMECs was detected by qRT-PCR. VEGFA protein expression levels were analyzed by Western blot and quantified (**i**). ** *p* < 0.01; *** *p* < 0.001, *p* > 0.05 was considered as not significant (ns).

Rescue assays were performed to explore the role of miR-135a-5p and whether miR-135a-5p regulated ABCB1 to affect the function of MMECs. As shown in Figure 9a–c, qRT-PCR analysis demonstrated that miR-135a-5p was upregulated in the miR-135a-5p mimic group (*p* < 0.001), and showed no significant alteration with the co-transfection of oe-ABCB1, while ABCB1 was downregulated in the miR-135a-5p mimic group (*p* < 0.001), and its expression was recovered by cotransfecting oe-ABCB1 in MMECs (*p* < 0.001). We also found that the concentration of DCA and LCA was increased in response to miR-135a-5p overexpression (*p* < 0.001), which was significantly reversed by upregulating ABCB1 (*p* < 0.001) (Figure 9d,e). Meanwhile, we demonstrated that ABCB1 overexpression rescued the upregulation in Tnf-α levels and reduction in Enos, Sod2, and Vegfa levels (*p* < 0.001) (Figure 9f–i).

### 2.9. Effects of CM from miR-135a-5p and Abcb1-Overexpressed MMECs on C2C12 Cells

**Figure 10:**
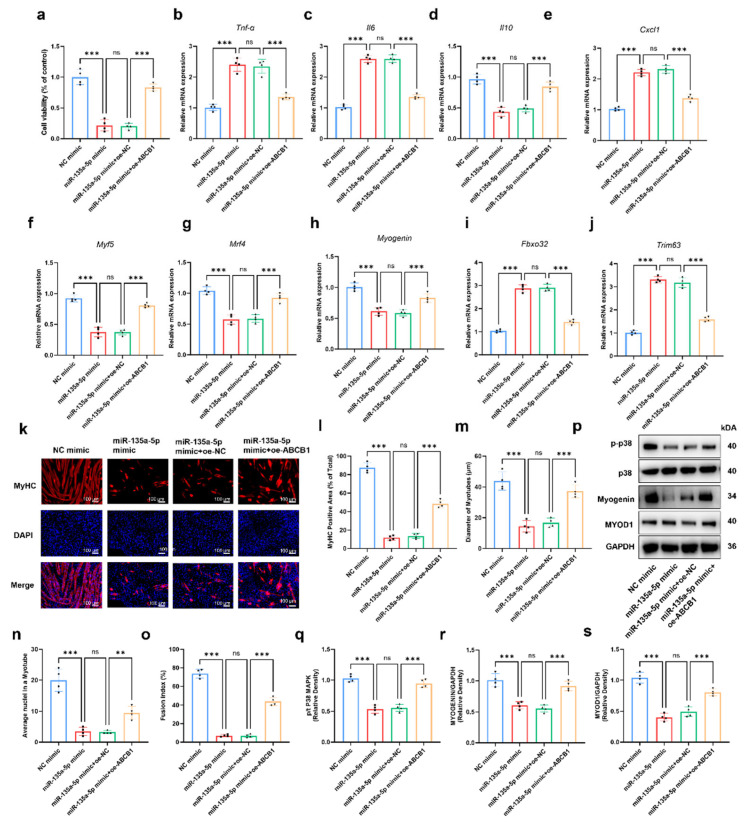
Effects of CM from miR-135a-5p and *Abcb1*-overexpressed MMECs on C2C12 cells. C2C12 cell viability was assessed by CCK-8 assays across groups (**a**). Expression of inflammatory factors *Tnf-α* (**b**) *Il6* (**c**), *Il10* (**d**), and *Cxcl1* (**e**) in myotubes treated with CM from each group was detected using qRT-PCR. Expression of myogenic key genes *Myf5* (**f**), *Mrf4* (**g**), *Myogenin* (**h**), *Fbxo32* (**i**), and *Trim63* (**j**) was also analyzed by qRT-PCR. Representative images of immunofluorescence staining of MyHC and DAPI staining in C2C12-derived myotubes treated with CM from transfected MMECs (**k**). Scale bar = 100 μm; red: MyHC, blue: nuclei. Measured and statistically analyzed: MyHC-positive area proportion (**l**), myotube diameter (**m**), average nuclei number per myotube (**n**), and myotube fusion index (**o**). Protein expression levels of phospho-p38 (p-p38), total p38 MAPK, MYOGENIN, and MYOD1 were assessed by Western blot (**p**) with quantification (**q**–**s**). *** *p* < 0.001, ** *p* > 0.01, *p* > 0.05 was considered as not significant (ns).

To validate whether miR-135a-5p regulates C2C12 cells through ABCB1 in MMECs, we performed rescue experiments. C2C12 myoblasts were incubated with CM from MMECs transfected with the NC mimic, miR-135a-5p mimic, or co-transfected with the miR-135a-5p mimic and oe-ABCB1. CCK-8 assays showed that C2C12 viability was significantly reduced in the miR-135a-5p mimic group compared to controls, and this effect was significantly reversed in the miR-135a-5p mimic + oe-ABCB1 group (Figure 10a). In C2C12-derived myotubes, *Tnf-α*, *Il6*, and *Cxcl1* expression was increased in the miR-135a-5p mimic group, but was significantly reduced in the miR-135a-5p mimic + oe-ABCB1 group; *Il10* expression showed the opposite alteration (Figure 10b–e). Similarly, the reduced expression of myogenic genes (*Myf5*, *Mrf4*, *Myogenin*) in the miR-135a-5p mimic group was rescued by *Abcb1* overexpression (Figure 10f–h), and the elevated atrophy-related genes *Fbxo32* and *Trim63* were significantly decreased in the miR-135a-5p mimic + oe-ABCB1 group (Figure 10i,j).

Immunofluorescence staining showed that CM from the miR-135a-5p mimic group significantly reduced the MyHC-positive area, myotube diameter, nuclei per myotube, and fusion index (*p* < 0.001), all of which were restored in the miR-135a-5p mimic + oe-ABCB1 group (Figure 10k–o).

Finally, the phosphorylation of p38 MAPK and protein levels of MYOGENIN and MYOD1 were downregulated in the miR-135a-5p mimic group, and significantly elevated in the miR-135a-5p mimic + oe-ABCB1 group (*p* < 0.001) (Figure 10p–s). These results confirm that miR-135a-5p impairs myoblast function by suppressing ABCB1 in MMECs.

## 3. Discussion

Sarcopenia is a complex geriatric syndrome with limited therapeutic options, largely due to our incomplete understanding of the cross-talk between the systemic environment and local muscle niches. For example, the role of the local microvascular environment remains poorly defined. Our study identifies a novel ‘bile acid–MMEC–muscle’ axis in the pathogenesis of sarcopenia. We demonstrate that the aging-related downregulation of the ABCB1 transporter in muscle microvascular endothelial cells (MMECs) is strongly associated with local bile acid accumulation. Supported by our in vitro mechanistic assays, we propose that this localized metabolic shift can trigger endothelial dysfunction and subsequently impair myoblast proliferation and differentiation.

To faithfully recapitulate the pathological progression of clinical sarcopenia, we utilized a naturally aged mouse model. It is noteworthy that while the sarcopenic mice in our model exhibited severe functional declines—as evidenced by significantly reduced grip strength and exercise capacity—the gross weight of the gastrocnemius muscle did not show a statistically significant reduction. This phenotype aligns with the well-documented concept of aging-related “dynapenia” [37], where the deterioration of muscle strength and functional capacity frequently precedes the absolute loss of macroscopic muscle mass [38,39]. Furthermore, this apparent discrepancy in gross weight is mechanistically explained by our histological findings. Although the individual functional myofibers underwent profound atrophy (significantly reduced cross-sectional area), this loss of contractile tissue was accompanied by a substantial expansion of the interstitial space due to fat infiltration (myosteatosis) and fibrotic tissue accumulation. Consequently, the accumulation of these non-contractile tissues compensates for the lost myofiber volume, maintaining the gross muscle weight while severely compromising muscle quality and mechanical function.

Within this deteriorated skeletal muscle microenvironment, analysis of bile acid concentrations in sarcopenic mice revealed significantly elevated levels of two major secondary bile acids—DCA and LCA—with higher concentrations observed in groups exhibiting lower muscle mass. Yasuyuki Tamai et al. [40] have reported increased serum total secondary bile acids in patients with low muscle mass. Similarly, Bénard Aliwa et al. [16] have demonstrated elevated serum secondary bile acids (DCA, LCA), DCA/Cholic Acid (CA), and LCA/Chenodeoxycholic Acid (CDCA) in cirrhotic patients with sarcopenia versus non-sarcopenic counterparts. Our results align with these findings. While current research predominantly focuses on systemic effects via FXR and TGR5 [41,42], including pathways involving FGF15/19-ERK [43,44] and TGR5-AKT/CaN signaling [18,45], our study shifts the focus to a previously understudied domain: the impaired bile acid efflux capacity in MMECs within the muscle microenvironment. In contrast, our study reveals that impaired muscle function in sarcopenic mice is associated with reduced *Abcb1* expression and diminished bile acid efflux capacity in MMECs—a previously understudied domain.

Our previous single-nucleus RNA sequencing (snRNA-seq) analysis of quadriceps femoris from sarcopenia patients revealed differential expression of genes—Adenosine Triphosphate (ATP), low-density lipoprotein receptor (LDLR), Multidrug Resistance Protein 1 (MDR1/ABCB1)—in MMECs versus healthy controls. Altered ATP may relate to mitochondrial metabolism [46], while LDLR changes could associate with muscular lipid infiltration [47]. Although sarcopenia research predominantly focuses on myofibers, MMECs play critical roles in angiogenesis, muscle regeneration, and inflammatory cell recruitment [48]. Trajectory analysis of denervated gastrocnemius in mice showed pronounced alterations in type I/II myofibers, followed by fibro-adipogenic progenitors and endothelial cells [49]. Timmerman et al. have demonstrated that MMEC functionality crucially regulates skeletal muscle protein synthesis, modulated by aging, inflammation, and exercise [50]. In aged mice, most apoptotic nuclei belonged to endothelial and satellite cells, with endothelial apoptosis preceding muscle atrophy [51].

Beyond being simple conduits, MMECs influence myoblast function through the secretion of lactate and regulatory factors like VEGFA and eNOS [52,53,54]. Additional studies indicate that autophagy mediates skeletal muscle regeneration independently of angiogenesis [55]. Collectively, these findings confirm endothelial cells influence skeletal muscle development and regeneration through diverse mechanisms. Our study highlights the critical role of MMEC-mediated bile acid efflux in sarcopenia, demonstrating that ABCB1 deficiency disrupts the local metabolic–vascular balance. Mechanistically, we found that pharmacological inhibition or genetic knockdown of *Abcb1* in MMECs significantly impairs their bile acid efflux capacity. Crucially, conditioned medium from these efflux-deficient MMECs acted in a paracrine manner to suppress C2C12 myoblast viability and S-phase proliferation, while simultaneously blunting myogenic differentiation—evidenced by the downregulation of key regulators (Myf5, MyoG, MyoD1) and the induction of atrophy markers (Fbxo32, Trim63). Conversely, restoring *Abcb1* expression via rescue experiments effectively reversed this pro-inflammatory and anti-myogenic secretome, restoring the regenerative potential of the muscle microenvironment. These results demonstrate that MMECs with impaired bile acid efflux enhance inflammatory responses and disrupt growth/differentiation in myoblasts—aligning with the established focus on chronic inflammation in sarcopenia pathogenesis [56,57]. Morphological analysis through immunofluorescence further confirmed that ABCB1-deficient MMEC-conditioned medium severely impairs myotube maturation, as evidenced by significant reductions in MyHC-positive area, myotube diameter, and the fusion index. Given that the conditioned medium was the sole experimental variable, we attribute these myogenic defects to the altered MMECs secretome—specifically the aberrant elevation of pro-inflammatory cytokines and the depletion of critical vasoprotective and antioxidant factors, including VEGFA, eNOS, and SOD2. These results underscore that the loss of ABCB1 transforms MMECs from a supportive niche into a source of paracrine stress, directly compromising the regenerative capacity of the skeletal muscle microenvironment in sarcopenia. Current evidence confirms VEGFA’s critical role in angiogenesis and muscle regeneration [58], while eNOS-derived NO and SOD2 maintain redox balance, exert anti-apoptotic effects, and promote vasodilation [59,60]. Their reduction directly compromises muscle repair and maintenance capabilities.

Integrating these mechanisms, Valspodar/Si-ABCB1 treatment impairs bile acid efflux in MMECs, leading to bile acid accumulation that disrupts endothelial tight junctions [61], increases microvascular permeability, reduces angiogenesis [26], promotes inflammatory factor diffusion into the muscle microenvironment [41], and diminishes myoblast nutritional support via microcirculation dysfunction. Thus, MMECs are not merely conduits but active regulators of the muscle microenvironment. Their bile acid efflux impairment—causing autodamage—acts as a significant contributing factor mediating bile acid-induced myoblast dysfunction. Extrapolating from these local microenvironmental findings, it is intriguing to hypothesize whether MMECs might serve as terminal effectors in the broader systemic ‘gut–muscle axis.’ However, defining this systemic cross-talk fully remains beyond the scope of the present study and warrants future investigation.

Intriguingly, Western blot analysis revealed reduced phospho-p38 MAPK levels in myoblasts treated with CM from the Valspodar Group and Si-ABCB1 Group. As a key regulator of inflammation, activated p38 MAPK promotes inflammatory responses by facilitating pro-inflammatory cytokine production and inhibiting neutrophil apoptosis [62]. Audrey E. Brown et al. [63] have observed significantly increased p38 MAPK activation in myotubes from insulin-resistant diabetic patients, correlating with upregulated inflammatory gene expression and identifying p38 MAPK as a critical modulator of pro-inflammatory transcriptomes. Paradoxically, our data showed enhanced inflammation alongside decreased phospho-p38 MAPK in myotubes treated with CM from the Valspodar Group and Si-ABCB1 Group. This apparent contradiction may arise from context-dependent roles of p38 MAPK—during active differentiation, myoblast proliferation can trigger p38 MAPK activation [64], while in skeletal muscle development, p38 MAPK is essential for early differentiation stages [65], and potentially upregulates MyoD and Myogenin expression [66].

P-gp (ABCB1) is a primary member of the ATP-binding cassette (ABC) transporter superfamily [22]. While extensively studied for its role in tumor multidrug resistance (MDR) [23], ABCB1 is equally critical for transporting endogenous substrates, including cholesterol [24,67,68], bile acids [69], and amyloid-β (Aβ) [70], thereby maintaining cellular and microenvironmental homeostasis. Similarly to the pathogenic role of ABC transporter deficiency in diabetic renal injury [68] and Alzheimer’s disease [71], we propose that impaired ABCB1-mediated transport disrupts the skeletal muscle niche. Unlike the liver-specific patterns observed in cancer cachexia [20], our findings specifically link aging-related sarcopenia to the downregulation of ABCB1 in both mice and murine MMECs. Our in vivo and in vitro experiments demonstrated that ABCB1-mediated bile acid transport in endothelial cells was closely related to muscle mass and function. Interestingly, our comprehensive transporter profiling extends this narrative by demonstrating a striking tissue- and disease-specific phenomenon—while BSEP and MRP1-4 remain unaltered in the aged skeletal muscle microenvironment, ABCB1 is highly and specifically downregulated.

Regarding the downregulation of ABCB1 in sarcopenic mice, potential mechanisms include the following: First, the aging process may directly involve decreased *Abcb1* expression [72]. Additionally, aging-associated alterations such as DNA methylation changes [73], histone modification processes [74], and altered miRNA expression profiles [36,75] may affect ABCB1 expression. We have explored the potential miRNAs that might regulate ABCB1 expression in sarcopenia progression. MiR-135a-5p has been demonstrated to be upregulated in muscle tissues of sarcopenic mice, which was consistent with the previous research [76,77]. *Abcb1* was directly targeted by miR-135a-5p in MMECs. Rescue assays further indicated that miR-135a-5p inhibits bile acid efflux and myotube differentiation by regulating *Abcb1*. However, in our study, sarcopenic mice were modeled using naturally aging mice to better reflect the natural progression of aging-related sarcopenia in patients. A limitation is the inability to exclude the confounding effects of age. Therefore, we established two sarcopenic mouse groups (N = 16) and further subdivided them into two subgroups (Group A and B) based on SMD measured by Micro-CT. Consequently, the comparison between Group A and B eliminates the confounding effects of aging. Thus, the downregulation of ABCB1 in Sarcopenia Group B mice (which exhibited poorer muscle function) may involve other mechanisms. Inflammatory factors and oxidative stress may regulate *Abcb1* expression [78]. Wolfgang W. Walther et al. have identified nuclear factor kappa-B (NF-κB)/p65 as a key regulator of *Abcb1* expression; in colon cancer cells, TNF-α downregulates multidrug resistance-related genes including *Abcb1* [79]. Skeletal muscle in aging-related sarcopenia exhibits a chronic inflammatory state [80], which was further confirmed by enhanced inflammatory factor expression in our cell experiments. This may be associated with ABCB1 downregulation.

Certain limitations in this study should be acknowledged. First, given the inherent differences between murine and human bile acid profiles, clinical cohort studies are required to fully extrapolate these findings to human sarcopenia. Second, while the gastrocnemius serves as a robust representative model for aging-related muscle loss, future studies incorporating diverse muscle types could provide a more comprehensive overview of whole-body skeletal muscle alterations. Third, although our in vitro conditioned media assays successfully demonstrated critical MMEC–myoblast crosstalk, they cannot fully recapitulate the complexity of the in vivo physiological bile acid pool or upstream microbiome dynamics. Finally, the precise extracellular concentrations of the secreted mediating factors in the conditioned medium were not directly quantified via protein assays (e.g., ELISA). However, the proposed paracrine regulation is strongly supported by the consistent intracellular protein validation in source MMECs (e.g., VEGFA) and the corresponding downstream target gene expression changes in recipient C2C12 cells. Further in vivo investigations and direct secretome profiling are warranted to fully map this “BA metabolism–MMEC–skeletal muscle axis”.

In conclusion, our study identifies a novel “BA metabolism–MMEC–skeletal muscle axis” in the pathogenesis of sarcopenia. We demonstrate that altered secondary bile acid profiles within the skeletal muscle microenvironment strongly correlate with impaired muscle function and reduced endothelial *Abcb1* expression in naturally aged mice. Mechanistically, the upregulation of miR-135a-5p in sarcopenic muscle specifically targets and downregulates *Abcb1* in MMECs. This suppression disrupts local BA efflux, leading to endothelial dysfunction that critically impairs myoblast viability and myogenic differentiation, a pathological process that can be effectively reversed by restoring *Abcb1* expression. Ultimately, these findings highlight that modulating local BA metabolism and preserving ABCB1 functionality in the microvascular endothelium represent promising therapeutic strategies to counteract aging-related muscle loss.

## 4. Materials and Methods

### 4.1. Animals and Experimental Design

Sixteen 24-month-old and eight 4-month-old male *C57BL/6J* mice were procured from Beijing Vital River Laboratory Animal Technology Co., Ltd. (Beijing, China) All mice were housed under controlled conditions at 22 °C with a 12 h light/dark cycle and provided ad libitum access to food and water. After a one-week acclimatization period, subsequent assessments were conducted. In accordance with established sarcopenia modeling protocols [34], naturally aged mice served as the sarcopenic animal model. Based on age, mice were allocated into the Sarcopenia Group (n = 16) and Control Group (n = 8, SMD = 146.36 ± 15.82 HU). SMD was defined as the mean Hounsfield Unit (HU) value of the skeletal muscle. To further control the variability in aging mice and distinguish the severity of sarcopenia, sarcopenic mice were further stratified into Sarcopenia Group A (n = 8, SMD = 126.89 ± 7.31 HU) and Sarcopenia Group B (n = 8, SMD = 94.96 ± 10.67 HU) with the median SMD value of the entire Sarcopenia Group (SMD = 115.02 HU) as the cutoff. The SMD difference between Group A and Group B was statistically significant (*p* < 0.0001). The sample size (n = 8 per group) was determined based on established protocols and standard practices widely accepted in murine models for sarcopenia research [81,82]. To retrospectively validate the statistical robustness of our sample size, a post hoc power analysis was conducted based on our primary functional outcome (grip strength) using G*Power software (version 3.1). The analysis yielded a large effect size (d = 3.59), confirming that n = 8 animals per group provided >99.9% of the statistical power needed to detect phenotypic differences at a two-sided α level of 0.05. Mice maintained their dietary regimens throughout body weight, body length, and grip strength measurements. Subsequently, after a 12–14 h fasting period, serum and muscle tissues were collected.

To evaluate the effects of *Abcb1* overexpression on sarcopenic mice in vivo, the adeno-associated virus serotype 9 (AAV9) that overexpressed the Abcb1 gene was constructed by GeneChem (Shanghai, China). Sarcopenic mice (24-month-old, SMD = 112.83 ± 10.33 HU) were randomly divided into three groups: the Sarcopenia Group, Sarcopenia + oe-NC Group, and Sarcopenia + oe-ABCB1 Group (n = 8/group). The uninjected naturally aged mice served as the baseline disease model (referred to as the ‘Sarcopenia Group’). For viral interventions, the sarcopenic mice were randomly allocated into two respective groups: the ‘Sarcopenia + oe-NC’ group, which received intramuscular injections of a negative control AAV), and the ‘Sarcopenia + oe-ABCB1’ group, which received intramuscular injections of an AAV specifically engineered to overexpress ABCB1. Mice were anesthetized with isoflurane, and a small incision was made into the right gastrocnemius muscle. Mice in the Sarcopenia + oe-NC Group and Sarcopenia + oe-ABCB1 Group were injected with 1 × 10^11^ vg particles of the AAV9 carrying oe-NC or oe-ABCB1 vectors into gastrocnemius muscle using a 31G Hamilton syringe. We utilized a multipoint injection technique (three distinct sites within the right gastrocnemius muscle) to ensure optimal tissue coverage and transduction uniformity. Mice in the Sarcopenia Group only received incisions without any injection. Mice were subjected to grip strength measurements after 4 weeks. After fasting for 12–14 h, the muscle tissues were harvested for further analysis. This study complied with all applicable international, national, and institutional guidelines for animal care and use. All animal procedures received formal ethical approval (Approval No. SZHY2024081501) from the Institutional Animal Care and Use Committee (IACUC) of Shouzheng Hongyao (Wuhan) Biotechnology Co., Ltd. (Wuhan, China) Furthermore, to minimize animal suffering during invasive procedures (e.g., AAV intramuscular injections), mice were fully anesthetized using continuous isoflurane inhalation. For postoperative analgesia and care, the mice received a subcutaneous injection of meloxicam (5 mg/kg) immediately following the procedure and once daily for three consecutive days, accompanied by daily monitoring for any signs of pain or distress.

### 4.2. Grip Strength Test

Grip strength was assessed using a Grip Strength Meter (SA 415, SANS Biotechnology, Nanjing, China). Mice were positioned on the grid with all four limbs gripping the mesh, ensuring their body remained parallel to the grid surface. The tail was gently grasped and pulled backward at a constant rate until the mouse released the grid. Each mouse underwent three trials with 10 min intervals between measurements. Grip duration (s) and peak force (N) were recorded automatically, and the mean grip strength value was calculated.

### 4.3. Micro CT

All *C57BL/6J* male mice were scanned using Bruker Micro-CT Skyscan 1276 system (Kontich, Belgium). Scan settings were as follows: voxel size 35 μm medium resolution, 85kV, 200 uA, 1 mm Al filter, and integration time 131 ms. Analysis was performed using the manufacturer’s evaluation software. Reconstruction was accomplished by NRecon (version 1.7.4.2). 3D images were obtained from contoured 2D images by methods based on distance transformation of the grayscale original images (CTvox; version 3.3.0). 3D and 2D analysis of ROIs (Regions of Interest) were performed by two independent blinded researchers (intra-class correlation coefficient > 0.9) using CT Analyser (version 1.20.3.0) software to calculate Skeletal Muscle Density (SMD) and Skeletal Muscle Area (SMA) in Lumbar 3.

### 4.4. Bile Acid Concentration Test

Bile acid concentrations in muscle tissue were quantified using high-performance liquid chromatography–tandem mass spectrometry (HPLC-MS/MS) (Shimadzu LC20AD, Shimadzu, Kyoto, Japan; API 3200MD TRAP system, AB Sciex, Framingham, MA, USA). Muscle tissues were weighed (approximately 20 mg per sample), homogenized in ultrapure water, and centrifuged. A 50 μL aliquot of the supernatant was transferred to a new tube, added with a mixture of stable isotopically labeled internal standards (ISs). Subsequently, 150 μL of acetonitrile was added for protein precipitation. After vortex-mixing for 1 min, samples were centrifuged at 13,200 rpm for 4 min at 4 °C. The resulting supernatant was collected for analysis.

Subsequently, intracellular bile acid quantification was performed by using calibration curves as an external standard. A standard curve was prepared for each analysis. The calibration curve exhibited excellent linearity (R^2^ > 0.99). Liquid chromatography conditions [83,84]: an ACQUITY UPLC^®^ BEH C18 column (2.1 × 100 mm, 1.7 μm, Waters, Milford, MA, USA) was used, the injection volume was 5 μL, the column temperature was 40 °C, and the mobile phase was A-0.01% formic acid water (B-acetonitrile). The gradient elution conditions were 0~4 min, 25% B; 4~9 min, 25~30% B; 9~14 min, 30~36% B; 14~18 min, 36~38% B; 18~24 min, 38~50% B; 24~32 min, 50~75% B; 32~33 min, 75~90% B; and 33~35.5 min, 90~25% B. The flow rate was 0.25 mL/min. Mass spectrum conditions [83,84]: electrospray ionization (ESI) source and negative ionization mode. The ion source temperature was 500 °C, the ion source voltage was −4500 V, the collision gas was 6 psi, the curtain gas was 30 psi, and the atomizing gas and auxiliary gas were both 50 psi. Scans were performed using multiple reaction monitoring (MRM).

### 4.5. Hematoxylin and Eosin (H&E) Stains

Histological analysis was performed on gastrocnemius muscle samples fixed in 4% paraformaldehyde (PFA). Following confirmation of adequate fixation, tissues were trimmed, dehydrated through graded ethanol series, embedded in paraffin, sectioned, and stained with hematoxylin and eosin (H&E). Qualified sections were identified through microscopic examination. Using SlideViewer software(version 2.5), target tissue regions were selected for 400× magnification imaging, ensuring complete tissue occupation within the field of view with uniform background illumination. Acquired images were analyzed with standardized metric units (mm/μm). Five randomly selected muscle fiber diameters were measured per field. Total cross-sectional area (CSA) of muscle fibers per field was quantified, and total fiber count was recorded. Mean fiber area was calculated as total CSA divided by fiber count. Fiber density was determined as fiber count per unit area.

### 4.6. RNA Isolation, Reverse Transcription, and Quantitative Real-Time Polymerase Chain Reaction (qRT-PCR)

Total RNA was extracted from tissues or cells with TRIzol reagent (Invitrogen, Waltham, MA, USA, 15596026). cDNA was synthesized using a RevertAid First Strand cDNA Synthesis Kit (Thermo Fisher Scientific, Waltham, MA, USA, K1621), and qPCR was performed using Power SYBR Green PCR Master Mix (Applied Biosystems, Waltham, MA, USA, A25742). Gene expression was calculated relative to that of Gapdh. The sequences of primers are listed in Table 2.

### 4.7. Protein Isolation and Western Blot Analysis

Total protein was isolated from gastrocnemius muscle using radio immunoprecipitation assay buffer (50 mM Tris-HCl, pH 7.4, 150 mM NaCl, 2 mM EDTA, 0.1% SDS, 0.1% Triton X-100, and 0.5% deoxycholate) with protease inhibitor cocktail and phosphatase inhibitors. Proteins were extracted from cells using RIPA lysis buffer (Thermo Fisher Scientific, Waltham, MA, USA, 89900) containing protease and phosphatase inhibitors (Thermo Fisher Scientific, Waltham, MA, USA, 78440). Protein concentrations were determined using PierceTM BCA protein assay kit (Thermo Fisher Scientific, Waltham, MA, USA, 23227). Then samples were separated by 10% SDS polyacrylamide gel electrophoresis and transferred onto polyvinylidene difluoride membranes (Millipore, Burlington, MA, USA, IPVH00010). The membranes were blocked in 5% skim milk and incubated overnight at 4 °C with primary antibodies against ABCB1 (Proteintech Group, Inc., Rosemont, IL, USA, 22336-1-AP, 1:1000), VEGFA (Abcam, Cambridge, UK, ab46154, 1:1000), MYOGENIN (Proteintech Group, Inc., Rosemont, IL, USA, 26762-1-AP, 1:10,000), MYOD1 (Proteintech, 18943-1-AP, 1:5000), p38 MAPK (Cell Signaling Technology, Danvers, MA, USA, #8690, 1:1000), phospho-p38 MAPK (Cell Signaling Technology, Danvers, MA, USA, #4511, 1:1000), and Glyceraldehyde-3-Phosphate Dehydrogenase (GAPDH, Abcam, Cambridge, UK, ab8245, 1:5000). After incubation, membranes were washed and incubated in secondary antibody for 1 h at room temperature. The second antibodies were from ZSGB-Bio (Beijing, China, anti-rabbit: ZB-2301; at dilutions of 1:10,000). After washing the membrane, apply freshly prepared ECL mixture solution to the protein side for chemiluminescence detection. The gray values of the Western blotting bands were analyzed using Image-Pro Plus software (version 6.0, Media Cybernetics Inc., Rockville, MD, USA).

### 4.8. MMEC Culture and Interventions

Cryopreserved mouse skeletal muscle microvascular endothelial cells (MMECs) from single donors (iCell Bioscience Inc., Shanghai, China) were thawed and cultured in endothelial basal medium (iCell Bioscience Inc., Shanghai, China) supplemented with 5% fetal bovine serum, 1% penicillin–streptomycin, and 1% MMEC Growth Supplement, maintained at 37 °C under 5% CO_2_ with medium changes every 48 h. Subsequent experiments were initiated when cells reached approximately 80% confluency, and passage 3–5 for MMECs were used in this study. Cells were dissociated with 0.25% trypsin-EDTA for 60–90 s until cellular rounding was observed microscopically, after which trypsinization was terminated by adding complete MMEC medium followed by gentle pipetting, centrifugation, supernatant removal, and cell counting prior to seeding into 6-well plates (Corning Costar CLS3516) at 2 × 10^6^ cells/well in 2.5 mL medium. Cells were pre-cultured for 24 h under standard conditions (37 °C, 5% CO_2_) until 80% confluency was attained. Our transfection was performed using 50 nM small interfering RNA (si-RNA) complexed with Lipofectamine 3000 (Invitrogen, Waltham, MA, USA, L3000008), specifically employing validated ABCB1-targeting si-RNA (si-ABCB1: sense 5′-GAUUGCGUUUGGAGGACAA(dT)-3′, antisense 5′-UUGUCCUCCAAACGCAAUC(dT)-3′) and scrambled control si-RNA (si-CON: sense 5′-UUCUCCGACAGUGUCACGU(dT)-3′, antisense 5′-ACGUGACACUGUCGGAGAA(dT)-3′) from Tsingke (Beijing, China). For miR-135a-5p and rescue experiments, miR-135a-5p mimics, oe-ABCB1 vectors, and the corresponding negative controls (NC mimics/oe-NC) were synthesized by GenePharma (Shanghai, China). MMECs seeded in 6-well plates were subjected to different treatments according to the following groups: Valspodar Group (10 μM Valspodar), DMSO Group (0.008% DMSO), Si-ABCB1 Group (si-ABCB1 transfection), Si-CON Group (si-CON transfection), miR-135a-5p Mimic Group (miR-135a-5p mimic transfection), NC Mimic Group (NC mimic transfection), miR-135a-5p Mimic + oe-ABCB1 Group (co-transfection of miR-135a-5p mimics and oe-ABCB1 vectors), and miR-135a-5p Mimic + oe-NC Group (co-transfection of miR-135a-5p mimics and oe-NC vectors). After 24 h of incubation for the Valspodar and DMSO Groups, or 72 h for the Si-ABCB1/Si-CON and all miRNA transfection groups (including NC mimics, miR-135a-5p mimics, miR-135a-5p mimic + oe-ABCB1, and miR-135a-5p mimic + oe-NC), the culture medium was replaced, and cells were incubated with 100 μM DCA and 100 μM LCA for 30 min. Following bile acid incubation, cells were washed three times with ice-cold Hanks’ balanced salt solution (HBSS) to terminate the uptake, then subjected to efflux initiation via 37 °C HBSS incubation for 30 min; after aspirating medium and inverting plates on absorbent paper, select cells underwent three 1 min ice-cold phosphate-buffered saline (PBS) washes with gentle agitation, plates were placed on ice, cells scraped in chilled PBS using cell scrapers, collected into pre-chilled centrifuge tubes, pelleted by low-speed centrifugation, flash-frozen in liquid nitrogen for 1 min, and stored at −80 °C for intracellular bile acid quantification. Some cells were subjected to protein extraction in preparation for subsequent Western blot analysis. The remaining cells received fresh endothelial basal medium for 48 h culture, after which conditioned medium was collected, transferred to 15 mL tubes, and stored at −80 °C for C2C12 cell experiments.

### 4.9. C2C12 Cell Culture and Interventions

Cryopreserved C2C12 mouse myoblasts (iCell Bioscience Inc., Shanghai) from single donors (iCell Bioscience Inc., Shanghai) were thawed and cultured in myoblast basal medium (iCell Bioscience Inc.) supplemented with 5% fetal bovine serum, 1% penicillin–streptomycin, and 1% C2C12 Growth Supplement, and maintained at 37 °C under 5% CO_2_ with medium changes every 48 h. Subsequent experiments were initiated when cells reached approximately 80% confluency, and passage 3–5 for C2C12 cells were used in this study. Cells were dissociated with 0.25% trypsin-EDTA for 90–120 s until cellular rounding was observed microscopically, after which trypsinization was terminated by adding complete C2C12 medium followed by gentle pipetting, centrifugation, supernatant removal, and cell counting prior to seeding into multiwell plates (6-well: Corning Costar CLS3516; 12-well: CLS3513; 48-well: CLS3548; 96-well: CLS3599). Cells were pre-cultured for 24 h under standard conditions (37 °C and 5% CO_2_) until 80% confluency was attained. For experiments evaluating the effects of MMEC-derived conditioned medium on C2C12 cells, myoblasts were then treated with a 1:1 mixture of myoblast basal medium and CM collected from different MMEC experimental groups. These included CM from the Valspodar Group and DMSO Group (pharmacological inhibition), the Si-ABCB1 Group and Si-CON Group (genetic knockdown), and for rescue experiments, the miR-135a-5p mimics Group, NC mimics Group, miR-135a-5p mimic + oe-ABCB1 Group, and miR-135a-5p mimic + oe-NC Group. Cell counting was performed using a hemocytometer (Hausser Scientific, Horsham, PA, USA) on days 0, 2, 4, and 6. On day 4, cell viability was assessed with a Cell Counting Kit-8 (CCK-8, Solarbio CA1210) based on water-soluble tetrazolium salt (WST); 10 μL CCK-8 solution was added per well in 96-well plates followed by 2 h incubation, with absorbance at 450 nm measured using a Multiskan Skyhigh microplate reader (Thermo Fisher Scientific, Waltham, MA, USA). Viability percentage was calculated according to the formula Viability (%) = [(A_treatment − A_blank)/(A_basal − A_blank)] × 100, where A_blank represents absorbance from wells containing medium and CCK-8 without cells.

For differentiation assays, C2C12 cells seeded in 6-well plates were grown to 80% confluency and induced with a 1:1 mixture of myogenic differentiation medium (high-glucose DMEM (Dulbecco’s modified Eagle medium) with 2% horse serum and 1% penicillin–streptomycin) and MMEC CM from the four experimental groups. Media were refreshed daily during differentiation, with myotube fusion phenomena observed from day 3 onward. On day 6, cells were harvested for myotube differentiation assessment and mRNA and protein isolation.

### 4.10. Immunocytochemistry

Immunofluorescence analyses for proliferation and differentiation were performed on treated C2C12 cells and differentiated myotubes. On day 4, proliferating cells were quantified via detection of 5-ethynyl-2′-deoxyuridine (EdU) (Beyotime Biotechnology, Shanghai, China, C0071S) binding to actively replicating DNA by adding pre-warmed 20 μM EdU working solution (1:1 vol/vol) to achieve a 10 μM final concentration in 48-well plates, which was incubated for 2 h at 37 °C; subsequently, medium was aspirated, cells fixed with 4% PFA at 4 °C, washed, permeabilized, incubated with click reaction cocktail for 30 min (room temperature, dark), washed again, and counterstained with 4′,6-diamidino-2-phenylindole (DAPI) (Invitrogen, Waltham, MA, USA); immediate imaging via fluorescence microscopy (ZEISS, Oberkochen, Germany) enabled quantification of proliferative cells (percentage and total counts/field) using Image-Pro Plus 6.0 (Media Cybernetics Inc., Rockville, MD, USA).

For differentiation assessment, MyHC staining was conducted—4% PFA-fixed cells were washed, permeabilized, blocked with 1% bovine serum albumin (BSA) for 30 min, incubated overnight at 4 °C with anti-MyHC primary antibody (R&D Systems, Minneapolis, MN, USA, MAB4470, 1:30 in 1% BSA), washed, incubated for 1 h with secondary antibody (Alexa Fluor 568, anti-mouse, Thermo Fisher Scientific, Waltham, MA, USA, catalog no. A10037) and DAPI (1:1000), then imaged. Myotubes (defined as MyHC^+^ structures containing ≥2 nuclei) were analyzed. Myotube fusion index was quantified as the nuclear proportion within MyHC–immunopositive syncytial structures, defined as multinucleated cells containing two or more nuclei. Average myotube size were measured using Image-Pro Plus 6.0, with four random microscopic fields per group imaged at low and high magnification included in statistical analysis.

### 4.11. RNA Pull-Down Assay

The biotinylated probe for miR-582-5p (bio-miR-582-5p) and miR-135a-5p (bio-miR-135a-5p) was obtained from Ribobio (Guangzhou, China). MMECs were lysed and cell lysate were incubated with biotinylated probe or oligo probe overnight at 4 °C. Then streptavidin magnetic beads (Thermo Fisher Scientific, Waltham, MA, USA) were added and incubated at 4 °C for 4 h. After washing the beads with washing buffer, the RNA complexes bound to the beads were eluted and isolated using Trizol reagent (Invitrogen, Waltham, MA, USA) followed by qRT-PCR analysis.

### 4.12. Dual-Luciferase Reporter Assay

The wild-type (WT) or mutant fragment of ABCB1 3′UTR containing the putative binding sequence for miR-135a-5p or miR-582-5p was sub-cloned into the pMIR-REPORT vector (Ambion, Austin, TX, USA). MMECs were seeded into 24-well plates and co-transfected with luciferase reporter constructs, and miR-135a-5p mimics or NC mimics using Lipofectamine 3000. After 48 h, cells were harvested, and the relative luciferase activity was determined using a Dual-Luciferase Reporter System Kit (Promega, Madison, WI, USA). Firefly luciferase activity was normalized to Renilla luciferase activity.

### 4.13. Statistical Analysis

Statistical analyses were performed using SPSS software (version 27.0, IBM Corp., Armonk, NY, USA). Continuous variables are expressed as mean ± standard deviation (SD) in the parametric tests, and as the median (interquartile range 25–interquartile range 75) of the non-parametric tests. Variance homogeneity was evaluated with the F test between groups or the Brown–Forsythe test among 3 or more groups). For data that followed normal distribution and variance homogeneity, intergroup differences were assessed by Student’s *t*-test, while multigroup comparisons utilized one-way ANOVA followed by Tukey’s post hoc test. Otherwise, Welch’s *t*-test was used for 2-group comparisons when variances were unequal; for non-normally distributed data, the Mann–Whitney U analysis was used for 2-group comparisons; and the Kruskal–Wallis test followed by Dunnett’s post hoc test was used for comparisons among 3 or more groups. Correlation analyses employed Spearman’s rank correlation coefficient for non-normally distributed variables. Statistical significance was defined as *p* ≤ 0.05: * *p* < 0.05; ** *p* < 0.01; *** *p* < 0.001; and **** *p* < 0.0001.

## Figures and Tables

**Table 1:** Behavioral Tests and Gastrocnemius Weight Comparison Among Sarcopenia Group A, Sarcopenia Group B, and the Control Group.

	Control	Sarcopenia A	Sarcopenia B	*p* Value
Grasp Duration (s)	10.36 ± 0.73	8.08 ± 1.47	7.71 ± 1.83	0.002 **
Average Power of Gripping (g)	86.22 ± 3.20	75.07 ± 5.26	69.82 ± 5.61	<0.001 ***
Ratio of Gripping to Body Weight	3.03 ± 0.15	2.45 ± 0.18	2.30 ± 0.23	<0.001 ***
Weight of GAS (g)	0.13 ± 0.027	0.15 ± 0.025	0.13 ± 0.021	0.071
Ratio of GAS Weight to Body Weight	0.0045 ± 0.001	0.0050 ± 0.0009	0.0042 ± 0.0008	0.197

GAS: gastrocnemius muscle. ** *p* < 0.01; *** *p* < 0.001.

**Table 2:** Primer Sequences for mRNA Expression.

Gene Name	Gene ID	Primer	Sequence (5′-3′)
*Abcb1*	18671	Forward	GTGGGAACTCTGGCTGCTATTA
		Reverse	AACATGGCTCTTTTATCGGCCT
*Bsep*	27413	Forward	GGACAATGATGTGCTTGTGG
		Reverse	CACACAAAGCCCCTACCAGT
*Mrp1*	17250	Forward	CCTGGAGCTTGCTCACCTAAAG
		Reverse	TTCAAACTGCGTCCGGATG
*Mrp2*	12780	Forward	AACTGCCTCTTCAGAATCTTA
		Reverse	GCCAGCCACGGAACCAGCTGCT
*Mrp3*	76408	Forward	ATCACCATACACAACGGCACCTTC
		Reverse	TCCGAGTAAGGCAGACACCAGAG
*Mrp4*	239273	Forward	CATACCATTGGTTCCGCTCT
		Reverse	TGCATCAAACAGCTCCTGAC
*Myf5*	17877	Forward	TTTCGAGACGCTCAAGAGGT
		Reverse	CAGACAGGGCTGTTACATTCA
*Mrf4*	17878	Forward	ATTCTTGAGGGTGCGGATTTCCTG
		Reverse	AAGACTGCTGGAGGCTGAGGCATC
*Myogenin*	17928	Forward	AGGTGTGTAAGAGGAAGTCTGT
		Reverse	TTGGGGTTGAGCAGGGTG
*Fbxo32*	67731	Forward	GCAAACACTGCCACATTCTCTC
		Reverse	CTTGAGGGGAAAGTGAGACG
*Trim63*	433766	Forward	TGACCACAGAGGGTAAAG
		Reverse	TGTCTCACTCATCTCCTTCTTC
*Tnf-α*	21926	Forward	ATGTCTCAGCCTCTTCTCATTC
		Reverse	GCTTGTCACTCGAATTTTGAGA
*Il6*	16193	Forward	CTCCCAACAGACCTGTCTATAC
		Reverse	CCATTGCACAACTCTTTTCTCA
*Il10*	16153	Forward	CTCGTTTGTACCTCTCTCCG
		Reverse	ATCTCCCTGGTTTCTCTTCC
*Il1β*	16176	Forward	CAGGCAGGCAGTATCACTCATTG
		Reverse	CGTCACACACCAGCAGGTTATC
*Cxcl1*	14825	Forward	ACCCGCTCGCTTCTCTGT
		Reverse	CACCTTTTAGCATCTTTTGG
*Vegfa*	22339	Forward	AGGGCAGAATCATCACGAAGT
		Reverse	AGGGTCTCGATTGGATGGCA
*Enos*	18127	Forward	TGATGGCGAAGCGAGTGAAG
		Reverse	ACTCATCCATACACAGGACCC
*Sod2*	20656	Forward	GCTCCGGTTTTGGGGTATCTG
		Reverse	GCGTTGATGTGAGGTTCCAG
*Gapdh*	14433	Forward	TGTCAAGCTCATTTCCTGGT
		Reverse	TAGGGCCTCTCTTGCTCAGT

## Data Availability

The raw data supporting the conclusions of this article will be made available by the authors on request.

## Supplementary Materials

The following supporting information can be downloaded at: https://www.mdpi.com/article/10.3390/ijms27062649/s1.
