# Supplementary material for: Bile Acid Metabolism Affects Muscle Regeneration in Aging Skeletal Muscle in a Manner Associated with Regulation of ABCB1 Expression
Source: Int J Mol Sci. 2026 Mar 13;27(6):2649. doi: 10.3390/ijms27062649 (PMC13027205; doi:10.3390/ijms27062649)
Supplement: Supplementary file 1 [file ijms-27-02649-s001.zip › ijms-4162662-supplementary.pdf]

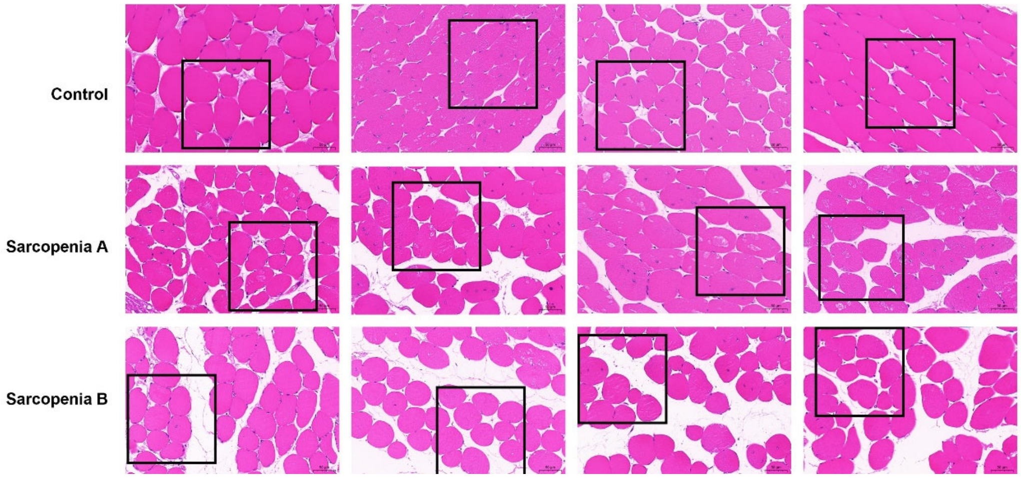

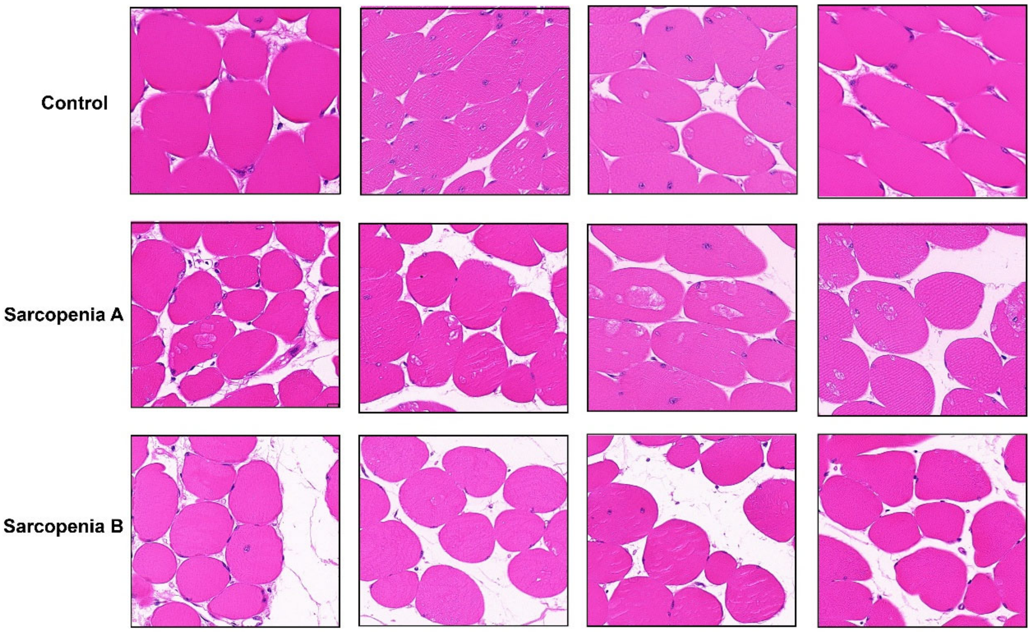

Supplementary Figure S1. Highly magnified representative H&E-stained images of the remaining skeletal muscle fibers from the Control, Sarcopenia A, and Sarcopenia B groups.

These magnified fields explicitly highlight the profound reduction in the CSA of individual myofibers in the sarcopenic mice, corroborating the quantitative data presented in main Figure 1b. The lower panels are highly magnified views of the regions enclosed by the corresponding black boxes in the original images.

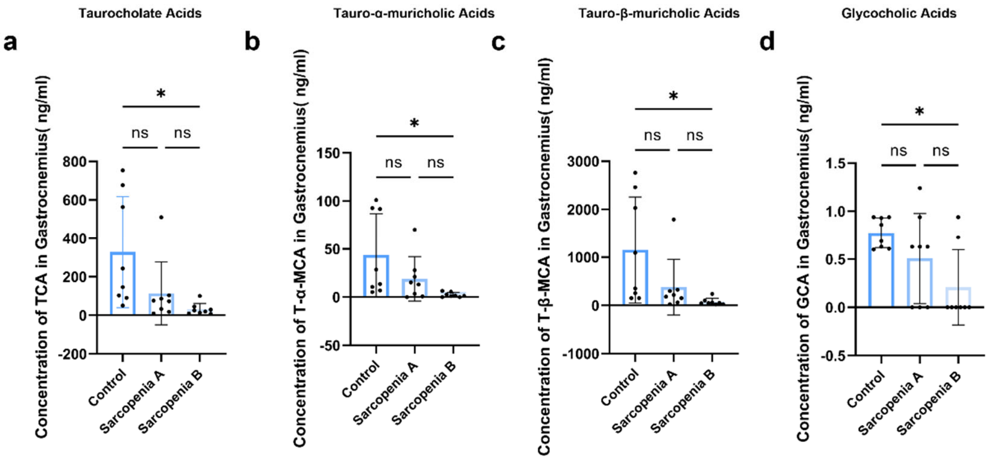

Supplementary Figure S2. Concentrations of Additional Bile Acids in the Muscle Tissue Microenvironment of the Control Group, Sarcopenia Group A, and Sarcopenia Group B.

Concentrations of taurocholic acid (TCA) (a), Tauro-α-muricholic acid (T-α-MCA) (b), tauro-β-muricholic acid (T-β-MCA) (c), and glycocholic acid (GCA) (d) in muscle tissue were measured using HPLC-MS/MS.

* $p < 0.05$, $p > 0.05$ was considered as not significant (ns).

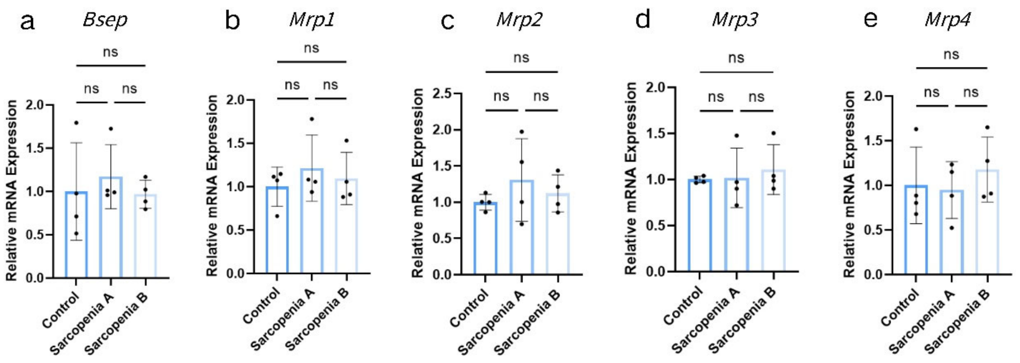

Supplementary Figure S3. The specific downregulation of Abcb1 among major efflux transporters in the skeletal muscle microenvironment.

The mRNA expression levels of other critical bile acid and multidrug efflux transporters, including *bile salt export pump (Bsep)* (a), *multidrug resistance-associated proteins 1-4 (Mrp1-4)* (b-e), in the quadriceps femoris muscle of the Control Group, Sarcopenia Group A, and Sarcopenia Group B mice were quantified by qRT-PCR. Quantitative analysis revealed no significant differences in the expression of these genes among the groups ($P > 0.05$). These results highlight the high specificity of Abcb1 downregulation in the pathogenesis of sarcopenia. Data are presented as mean ± SD (n = 4–5 per group). $p > 0.05$ was considered as not significant (ns).

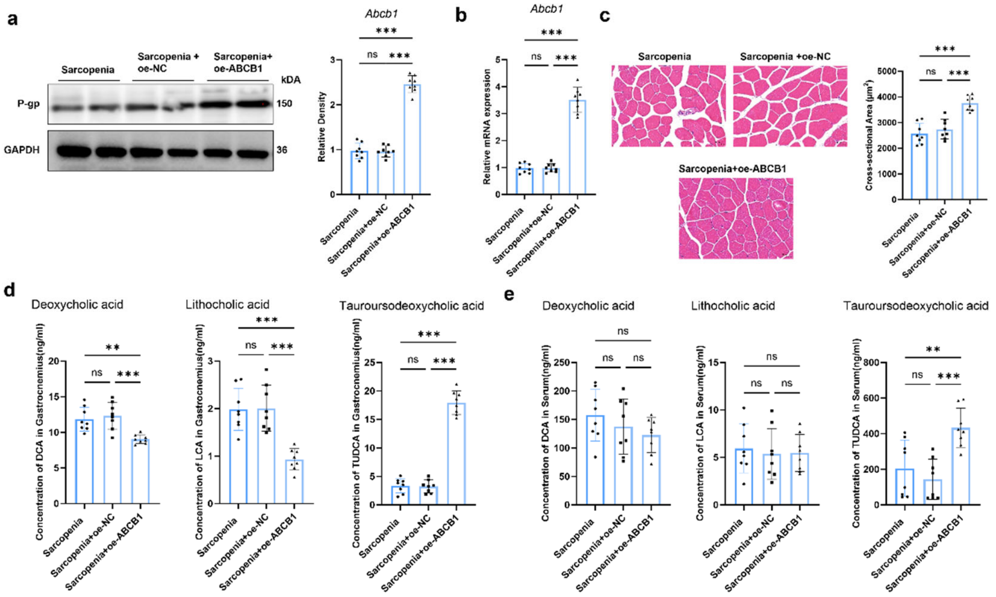

Supplementary Figure S4. Effects of adeno-associated virus serotype 9 (AAV9)-mediated Abcb1 overexpression on skeletal muscle CSA and bile acid concentrations in sarcopenic mice.

P-gp protein expression levels were detected by Western Blot analysis and quantified (a). *Abcb1* mRNA levels were measured by qRT-PCR analysis (b). Representative H&E stained cross sections of quadriceps femoris muscle from the Sarcopenia Group, and Sarcopenia + oe-NC Group B, and Sarcopenia + oe-ABCB1 Group of mice (scale bar: 50 μm), and quantification of CSA differences (c). Concentrations of DCA, LCA, and TUDCA in gastrocnemius muscle microenvironment (d) and serum (e) in each group were measured by HPLC-MS/MS.

** $p < 0.01$; *** $p < 0.001$, $p > 0.05$ was considered as not significant (ns).

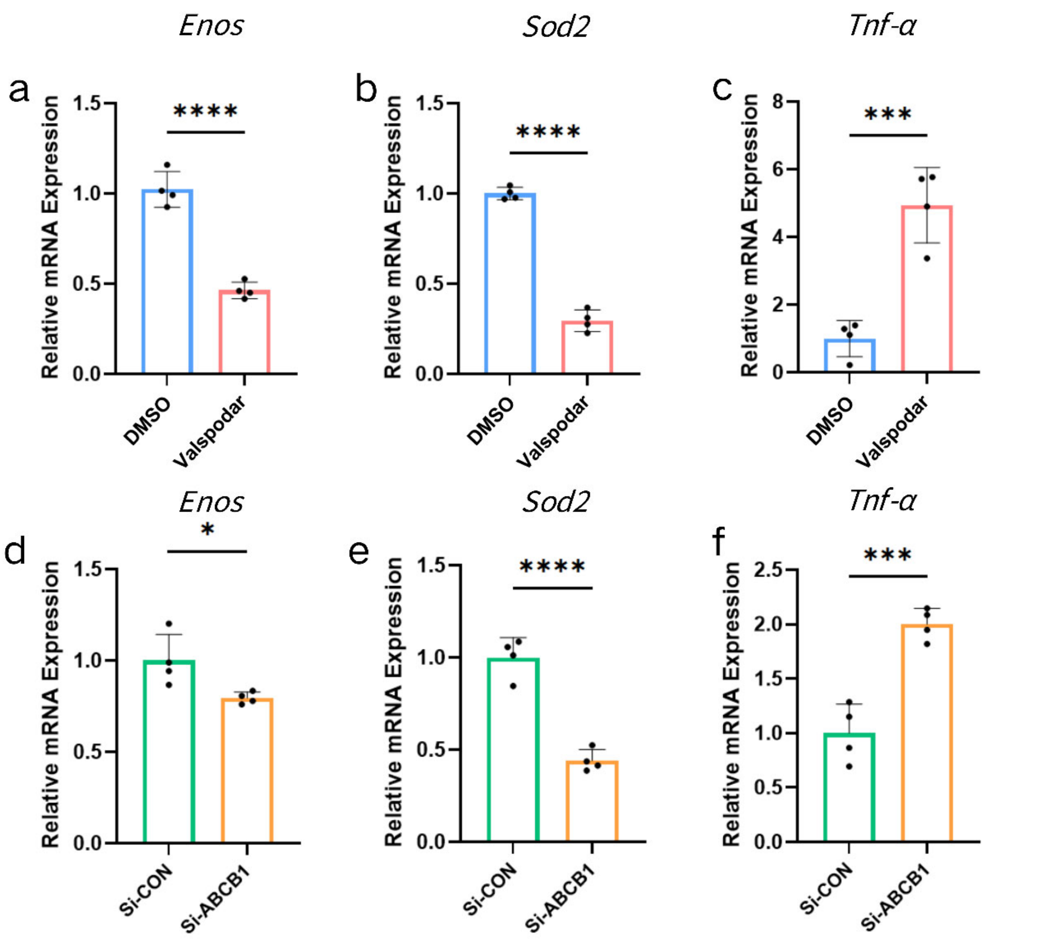

Supplementary Figure S5. Altered Expression of Endothelial Function Regulatory Genes in MMECs of the Valspodar Group and Si-ABCB1 Group.

Expression of *Enos* (a, d), *Sod2* (b, e) and *Tnf-α* (c,f) in MMECs was detected by qRT-PCR.

* $p < 0.05$; *** $p < 0.001$; and **** $p < 0.0001$.
